# Supplementary material for: Optical coherence tomography quantifies gradient refractive index and mechanical stiffness gradient across the human lens
Source: Commun Med (Lond). 2024 Aug 12;4:162. doi: 10.1038/s43856-024-00578-9 (PMC11319654; doi:10.1038/s43856-024-00578-9)
Supplement: Supplementary file 2 — Description of Additional Supplementary Files [file 43856_2024_578_MOESM2_ESM.pdf]

Description of Additional Supplementary Files

File name- Supplementary Data 1

File description- Supplementary Data 1 contains source data supporting figures 2, 4, 5.

File name- Supplementary Data 2

File description- The numerical data used to plot Figures 2 a-d and 4 a-h can be found in Supplementary Data 2.
