# Supplementary material for: Optical coherence tomography quantifies gradient refractive index and mechanical stiffness gradient across the human lens
Source: Commun Med (Lond). 2024 Aug 12;4:162. doi: 10.1038/s43856-024-00578-9 (PMC11319654; doi:10.1038/s43856-024-00578-9)
Supplement: Supplementary file 5 — Reporting Summary [file 43856_2024_578_MOESM5_ESM.pdf]

nature portfolio

Corresponding author(s): Sabine Kling

Last updated by author(s): May 7, 2024

Reporting Summary

Nature Portfolio wishes to improve the reproducibility of the work that we publish. This form provides structure for consistency and transparency in reporting. For further information on Nature Portfolio policies, see our Editorial Policies and the Editorial Policy Checklist.

Statistics

For all statistical analyses, confirm that the following items are present in the figure legend, table legend, main text, or Methods section.

n/a	Confirmed	
☐	☒	The exact sample size (*n*) for each experimental group/condition, given as a discrete number and unit of measurement
☐	☒	A statement on whether measurements were taken from distinct samples or whether the same sample was measured repeatedly
☐	☒	The statistical test(s) used AND whether they are one- or two-sided<br>*Only common tests should be described solely by name; describe more complex techniques in the Methods section.*
☒	☐	A description of all covariates tested
☐	☒	A description of any assumptions or corrections, such as tests of normality and adjustment for multiple comparisons
☐	☒	A full description of the statistical parameters including central tendency (e.g. means) or other basic estimates (e.g. regression coefficient) AND variation (e.g. standard deviation) or associated estimates of uncertainty (e.g. confidence intervals)
☐	☒	For null hypothesis testing, the test statistic (e.g. *F*, *t*, *r*) with confidence intervals, effect sizes, degrees of freedom and *P* value noted<br>*Give P values as exact values whenever suitable.*
☒	☐	For Bayesian analysis, information on the choice of priors and Markov chain Monte Carlo settings
☒	☐	For hierarchical and complex designs, identification of the appropriate level for tests and full reporting of outcomes
☒	☐	Estimates of effect sizes (e.g. Cohen's *d*, Pearson's *r*), indicating how they were calculated

Our web collection on statistics for biologists contains articles on many of the points above.

Software and code

Policy information about availability of computer code

Data collection	The driving software (research version) of the Anterion OCT device, Heidelberg Engineering, was used to collect the raw images.
Data analysis	Customized routines written in Matlab software were used for post-processing, which are available on Zenodo.

For manuscripts utilizing custom algorithms or software that are central to the research but not yet described in published literature, software must be made available to editors and reviewers. We strongly encourage code deposition in a community repository (e.g. GitHub). See the Nature Portfolio guidelines for submitting code & software for further information.

Data

Policy information about availability of data

All manuscripts must include a data availability statement. This statement should provide the following information, where applicable:

- Accession codes, unique identifiers, or web links for publicly available datasets
- A description of any restrictions on data availability
- For clinical datasets or third party data, please ensure that the statement adheres to our policy

The raw OCT data set cannot be publicly disclosed to safeguard the privacy of the study participants. Source data supporting the figures can be found in the supplementary data.

Human research participants

Policy information about studies involving human research participants and Sex and Gender in Research.

Reporting on sex and gender	Overall, a group of mixed sex was analysed. Consent has not been obtained to share data on an individual level.
Population characteristics	The data set contained participants of a wide age range with no known diseases related to the crystalline lens.
Recruitment	Previously collected data sets from our recent in vivo elastography studies in healthy participants (approved by the ethics committee of the ETH Zurich) and progressive keratoconus patients (approved by the cantonal ethics committee Zurich, number 2021-02275) were anonymized and re-examined with a focus on the crystalline lens. The only demographic parameter that was preserved was the age category of the person (intervals of 5 years). Exclusion criteria were any pre-existing corneal disease other than keratoconus, anatomical constraints which prevent the correct placement of the measurement devices, pregnancy, a minimal corneal thickness of less than 350 µm or inability to understand the nature of the study and/or give consent. The study was conducted in accordance with the Declaration of Helsinki.
Ethics oversight	cantonal ethics committee Zurich, number 2021-02275; ethics committee of the ETH Zurich

Note that full information on the approval of the study protocol must also be provided in the manuscript.

Field-specific reporting

Please select the one below that is the best fit for your research. If you are not sure, read the appropriate sections before making your selection.

☒ Life sciences ☐ Behavioural & social sciences ☐ Ecological, evolutionary & environmental sciences

For a reference copy of the document with all sections, see nature.com/documents/nr-reporting-summary-flat.pdf

Life sciences study design

All studies must disclose on these points even when the disclosure is negative.

Sample size	a previously collected data set was re-examined; thereof n=12 independent biological samples were included
Data exclusions	Data were excluded in case that 1) less than 95% of the lens thickness were visible in the scan, or 2) the measurement was corrupted by patient movement, including blinking
Replication	The assessment of pressure-induced strain in the lens has previously been observed by our group in a different set of participants. The assessment of micro-fluctuations of accommodation was repeated prospectively in six additional participants (for the purpose of an ongoing study). Therefore, replicability can be confirmed in both cases.
Randomization	not applicable
Blinding	Not relevant in this exploratory study design.

Reporting for specific materials, systems and methods

We require information from authors about some types of materials, experimental systems and methods used in many studies. Here, indicate whether each material, system or method listed is relevant to your study. If you are not sure if a list item applies to your research, read the appropriate section before selecting a response.

Materials & experimental systems

n/a	Involved in the study
☒	☐ Antibodies
☒	☐ Eukaryotic cell lines
☒	☐ Palaeontology and archaeology
☒	☐ Animals and other organisms
☒	☐ Clinical data
☒	☐ Dual use research of concern

Methods

n/a	Involved in the study
☒	☐ ChIP-seq
☒	☐ Flow cytometry
☒	☐ MRI-based neuroimaging
