# Supplementary material for: Optical coherence tomography quantifies gradient refractive index and mechanical stiffness gradient across the human lens
Source: Commun Med (Lond). 2024 Aug 12;4:162. doi: 10.1038/s43856-024-00578-9 (PMC11319654; doi:10.1038/s43856-024-00578-9)
Supplement: Supplementary file 6 — Supplementary information [file 43856_2024_578_MOESM6_ESM.pdf]

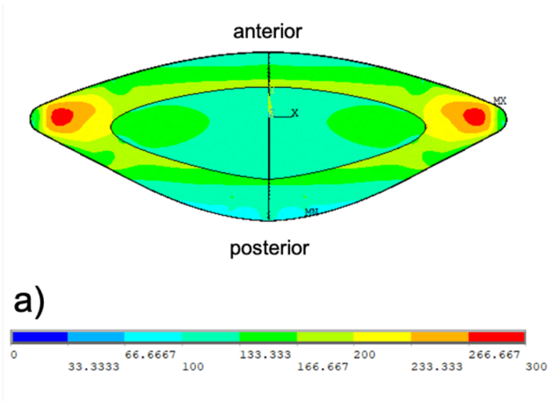

Supplementary Figure 1. Simulation results. Von Mises (A) stress and (B) strain distribution during full accommodation in a 32.5 year-old lens.
